# Small, Seeding-Competent Huntingtin Fibrils Are Prominent Aggregate Species in Brains of zQ175 Huntington’s Disease Knock-in Mice

**DOI:** 10.3389/fnins.2021.682172

**Published:** 2021-06-22

**Authors:** Franziska Schindler, Nicole Praedel, Nancy Neuendorf, Severine Kunz, Sigrid Schnoegl, Michael A. Mason, Bridget A. Taxy, Gillian P. Bates, Ali Khoshnan, Josef Priller, Jan Grimm, Marcel Maier, Annett Boeddrich, Erich E. Wanker

**Affiliations:** ^1^Neuroproteomics, Max Delbrück Center for Molecular Medicine in the Helmholtz Association, Berlin, Germany; ^2^Huntington’s Disease Centre, Department of Neurodegenerative Disease, UK Dementia Research Centre, UCL Queen Square Institute of Neurology, University College London, London, United Kingdom; ^3^Division of Biology and Biological Engineering, California Institute of Technology, Pasadena, CA, United States; ^4^Department of Psychiatry and Psychotherapy, Klinikum Rechts der Isar, Technical University of Munich, Munich, Germany; ^5^Charité–Universitätsmedizin Berlin and DZNE, Berlin, Germany; ^6^The University of Edinburgh, UK Dementia Research Institute, Edinburgh, United Kingdom; ^7^Neurimmune AG, Schlieren, Switzerland

**Keywords:** Huntington’s disease, aggregates, seeding, mHTT, zQ175, brain, protein misfolding, FRASE assay

## Abstract

The deposition of mutant huntingtin (mHTT) protein aggregates in neurons of patients is a pathological hallmark of Huntington’s disease (HD). Previous investigations in cell-free and cell-based disease models showed mHTT exon-1 (mHTTex1) fragments with pathogenic polyglutamine (polyQ) tracts (>40 glutamines) to self-assemble into highly stable, β-sheet-rich protein aggregates with a fibrillar morphology. HD knock-in mouse models have not been extensively studied with regard to mHTT aggregation. They endogenously produce full-length mHTT with a pathogenic polyQ tract as well as mHTTex1 fragments. Here, we demonstrate that seeding-competent, fibrillar mHTT aggregates can be readily detected in brains of zQ175 knock-in HD mice. To do this, we applied a highly sensitive FRET-based protein amplification assay that is capable of detecting seeding-competent mHTT aggregate species down to the femtomolar range. Furthermore, we show that fibrillar structures with an average length of ∼200 nm can be enriched with aggregate-specific mouse and human antibodies from zQ175 mouse brain extracts through immunoprecipitations, confirming that such structures are formed *in vivo*. Together these studies indicate that small, fibrillar, seeding-competent mHTT structures are prominent aggregate species in brains of zQ175 mice.

## Introduction

Huntington’s disease (HD) is a progressive neurodegenerative disorder that is characterized by motor, cognitive and psychiatric manifestations (Ross and Tabrizi, 2011; McColgan and Tabrizi, 2018). The disease is caused by a CAG repeat expansion encoding a polyglutamine (polyQ) tract within the N-terminus of huntingtin (HTT), a large α-helical protein (Guo et al., 2018), which plays a functional role in transcription regulation, autophagy and axonal transport processes (Saudou and Humbert, 2016).

Large neuronal intranuclear inclusion bodies (IBs) that contain mutant HTT (mHTT) protein aggregates, molecular chaperones and ubiquitin are the prominent hallmark of the pathology in HD patient brains (DiFiglia et al., 1997; Sieradzan et al., 1999), suggesting that misfolding and abnormal aggregation of mHTT may contribute to pathogenesis or even drive the disease process (Wanker et al., 2019). Besides nuclear IBs, however, also neuropil structures with mHTT aggregates and cytoplasmic IBs are commonly detected in neurons (Gutekunst et al., 1999), demonstrating that different types of mHTT aggregates are formed in HD patient brains that may have distinct roles in pathogenesis. This view is supported by studies in transgenic and knock-in (KI) HD mice, indicating that formation of neuropil mHTT aggregates is an early pathological event associated with axonal degeneration (Li et al., 2001; Tallaksen-Greene et al., 2005).

Our current understanding of HD is largely based on studies of mHTT-induced pathogenesis in transgenic and KI mouse models (Farshim and Bates, 2018). Among these, the KI mouse models carry expanded CAG repeats contained within the native murine *Htt* gene (Menalled et al., 2003; Heng et al., 2007). Thus, in comparison to transgenic models that were generated by inserting either a fragment or a full-length copy of the human *HTT* gene into the mouse genome (Mangiarini et al., 1996) they are closer to the situation in patients because they express the CAG mutation in the appropriate genetic context (Menalled et al., 2003; Farshim and Bates, 2018). The zQ175 KI line is a particularly useful model to study the molecular mechanisms of HD since it exhibits extensive behavioral, histopathological and molecular phenotypes reminiscent of human disease (Menalled, 2005; Heikkinen et al., 2012). Amongst the defects reported, zQ175 mice display behavioral deficits, especially in the dark phase of the diurnal cycle, from 4.5 months of age and overt rotarod impairments by 8 months of age (Menalled et al., 2012). Also, a progressive increase of nuclear and extranuclear inclusions with mHTT aggregates was observed in the striatum and cortex of zQ175 heterozygous mice (Carty et al., 2015), indicating that abnormal mHTT aggregation is associated with the appearance of disease symptoms in this model.

Currently, it remains unclear whether different types of mHTT aggregate species play a role in the observed disease process in zQ175 KI mice. Previous evidence has been obtained that N-terminal mHTT exon-1 (mHTTex1) fragments spontaneously self-assemble into stable β-sheet fibrillar structures *in vitro* and *in vivo* (Scherzinger et al., 1997, 1999; Waelter et al., 2001) and are also able to spread from cell to cell (Babcock and Ganetzky, 2015; Maxan et al., 2020). Whether such seeding- and spreading-competent structures are directly involved in pathogenesis is still a matter of debate (Hoffner and Djian, 2015). The formation of small seeding-competent mHTTex1 fibrils in adult neurons was recently shown to dramatically shorten the lifespan of HD transgenic flies (Ast et al., 2018), suggesting that similar structures may also contribute to pathogenesis in brains of zQ175 KI mice.

Here, we utilized a recently developed Förster resonance energy transfer (FRET)-based protein aggregate amplification assay (FRASE) (Ast et al., 2018) as well as various biochemical techniques to investigate whether seeding-competent, fibrillar mHTT aggregates are formed in brains of zQ175 mice. We found that such structures are detectable in brain extracts prepared from zQ175 KI mice but not in age-matched controls. Also, utilizing immunoprecipitation experiments, fibrillar, seeding-competent mHTT aggregates were enriched from zQ175 mouse brain extracts with various aggregate-specific mHTT antibodies, supporting our hypothesis that such structures are formed *in vivo*. Our findings will enable future studies to evaluate the effects of therapeutic molecules both on mHTT fibrillogenesis and disease symptoms in zQ175 KI mice.

## Results

### Preformed Recombinant mHTTex1 Fibrils Stimulate Ex1Q48-CyPet/-YPet Co-aggregation in FRASE Assays

**FIGURE 1:**
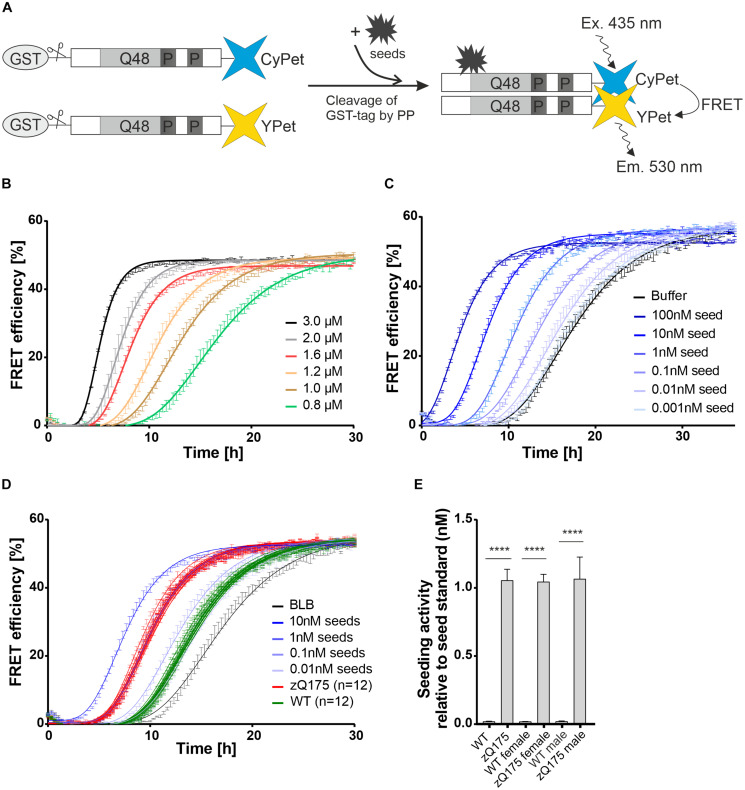
Establishment of FRET-based mHTT aggregate seeding assay (FRASE) for testing biological samples. **(A)** Schematic representation of FRASE. PP, PreScission Protease. **(B)** Time- and concentration-dependent co-aggregation of Ex1Q48-CyPet and Ex1Q48-YPet sensor proteins (1:1 mixture, 0.8–3.0 μM) monitored by repeated FRET measurements. FRET efficiency is displayed as mean ± SEM of technical triplicates. **(C)** Effect of different concentrations of preformed Ex1Q48 seeds on Ex1Q48-CyPet and Ex1Q48-YPet (1:1 mixture, 1.2 μM) co-aggregation. Data are mean ± SEM of technical replicates. **(D)** FRASE analysis of crude brain extracts prepared from 6-month-old WT and zQ175 mice. Whole hemispheres were used for preparation. Different concentrations of preformed sonicated recombinant Ex1Q48 aggregates were used as seed standard. 4 μg total mouse protein per replicate was applied. BLB, brain lysis buffer. Data are mean ± SEM of three technical replicates. Number of biological replicates is indicated. **(E)** Quantification of seeding activity in mouse brain extracts investigated in panel **(D)**. Seeding activities were normalized to the seed standard. The first bars (WT and zQ175 mice) represent all 12 biological replicates analyzed in panel **(D)**. Six of these mice were female and six were male. The following bars represent six biological replicates of female and male mice, respectively. Data are mean ± SEM of biological replicates. Statistical analysis: Unpaired *t*-test between WT and zQ175 mice (****, *p* value < 0.0001).

Previously, we have developed a FRET-based mHTT aggregate seeding assay (FRASE) that enables the quantification of mHTT seeding activity (HSA) in complex biosamples such as crude human brain extracts (Ast et al., 2018). For quantification of HSA, two purified glutathione S-transferase (GST)-tagged HTT exon-1 fusion proteins with 48 glutamines C-terminally fused to CyPet and YPet (GST-Ex1Q48-CyPet and -YPet) are utilized as reporter proteins (Figure 1A). They are soluble in aqueous solutions as long as they contain the N-terminal GST tag. When they are cleaved with PreScission protease (PP) the Ex1Q48-CyPet and -YPet fragments are released and they spontaneously form SDS-stable fibrillar co-aggregates after a lag phase of 8–10 h in a time-dependent manner. Their co-aggregation can be quantified by measuring FRET as the fluorescent tags CyPet and YPet are brought into close proximity when fibrillar mHTTex1 aggregates are formed (Figure 1A). Time-dependent co-aggregation can be accelerated by the addition of preformed fibrillar mHTT seeds, which function as templates for the conversion of the reporter proteins from a soluble into an aggregated state (Wagner et al., 2018). To benchmark the assay, control experiments with preformed fibrillar mHTTex1 seeds were performed. First, the recombinant fusion proteins GST-Ex1Q48-CyPet and -YPet were produced in *Escherichia coli* and purified to ∼90% homogeneity using glutathione agarose columns. Furthermore, GST-tagged HTTex1 fusion protein with 48 glutamines (GST-Ex1Q48) was produced and purified from *E. coli* protein extracts. This protein was later utilized for the production of recombinant seeds to stimulate the co-aggregation of the reporter proteins Ex1Q48-CyPet and -YPet in FRASE assays (Figure 1A). All purified recombinant fusion proteins were characterized by SDS-PAGE and Coomassie blue R staining. The fusion proteins GST-Ex1Q48-CyPet and -YPet migrated at ∼80 kDa in SDS-gels (Supplementary Figure 1A), while for the GST-Ex1Q48 fusion protein a molecular weight of ∼60 kDa was determined. The purified GST fusion proteins’ molecular mass corresponds to the molecular weights expected from their amino acid sequence.

To assess the aggregation propensity of the purified fusion proteins, we incubated different concentrations of GST-Ex1Q48-CyPet and -YPet (1:1 molar ratio) in 384-well plates with PP and quantified the time- and concentration-dependent appearance of FRET. We observed a steep increase in FRET efficiency after 4–10 h (Figure 1B), indicating that the released fragments Ex1Q48-CyPet and -YPet spontaneously form fibrillar mHTTex1 co-aggregates *in vitro*.

Next, preformed fibrillar Ex1Q48 aggregates were produced by cleavage and sonication of GST-Ex1Q48 fusion protein (Supplementary Figure 1B). We observed that the generated Ex1Q48 fibrils show a change in fluorescence upon addition of the amyloid dye thioflavin T (Supplementary Figure 1C), confirming that they consist of β-sheets (Wagner et al., 2018). Preformed seeds were then added to Ex1Q48-CyPet and -YPet co-aggregation reactions to assess their activity. We found a concentration-dependent shortening of the lag phase when preformed fibrillar Ex1Q48 seeds were added to reactions (Figure 1C), indicating that these structures potently stimulate Ex1Q48-CyPet and -YPet co-polymerization in cell-free assays. Thus, preformed Ex1Q48 fibrils can be utilized as positive control when complex biosamples with unknown concentrations of mHTT fibrils are analyzed for their HSA in FRASE assays.

### Detection of Seeding-Competent mHTT Aggregates in Brains of zQ175 Knock-in Mice Using FRASE Assays

Immunohistological investigations showed that IBs with mHTT aggregates are present in brains of 6-month-old zQ175 mice (Carty et al., 2015). We hypothesized that mHTT fibrils with seeding activity might also be detectable in brains of these mice. To address this question, we produced crude brain extracts from male and female heterozygous zQ175 and age-matched wild-type (WT) controls (six brains each) and quantified HSA using the established FRASE assay. All mouse brain samples were assessed on one 384-well plate together with different concentrations of preformed recombinant Ex1Q48 seeds as positive controls. Measurements were taken in three technical replicates. We observed similar HSA in all zQ175 brain samples, while HSA was not detected in brain extracts from age-matched control mice (Figures 1D,E). This indicates that fibrillar, seeding-competent mHTT aggregates are formed in brains of zQ175 mice but not in controls. Also, HSA was similar in brain extracts prepared from female and male zQ175 mice, indicating that sex does not significantly influence the formation of mHTT fibrils. Similar HSAs were determined when brain extracts prepared from 12-month-old zQ175 and wild-type control mice were analyzed with the FRASE assay (Supplementary Figures 2A,B).

### Detection of Large, SDS-Stable mHTT Aggregates in R6/2 and zQ175 Mouse Brains by SDS-PAGE and Immunoblotting

**FIGURE 2:**
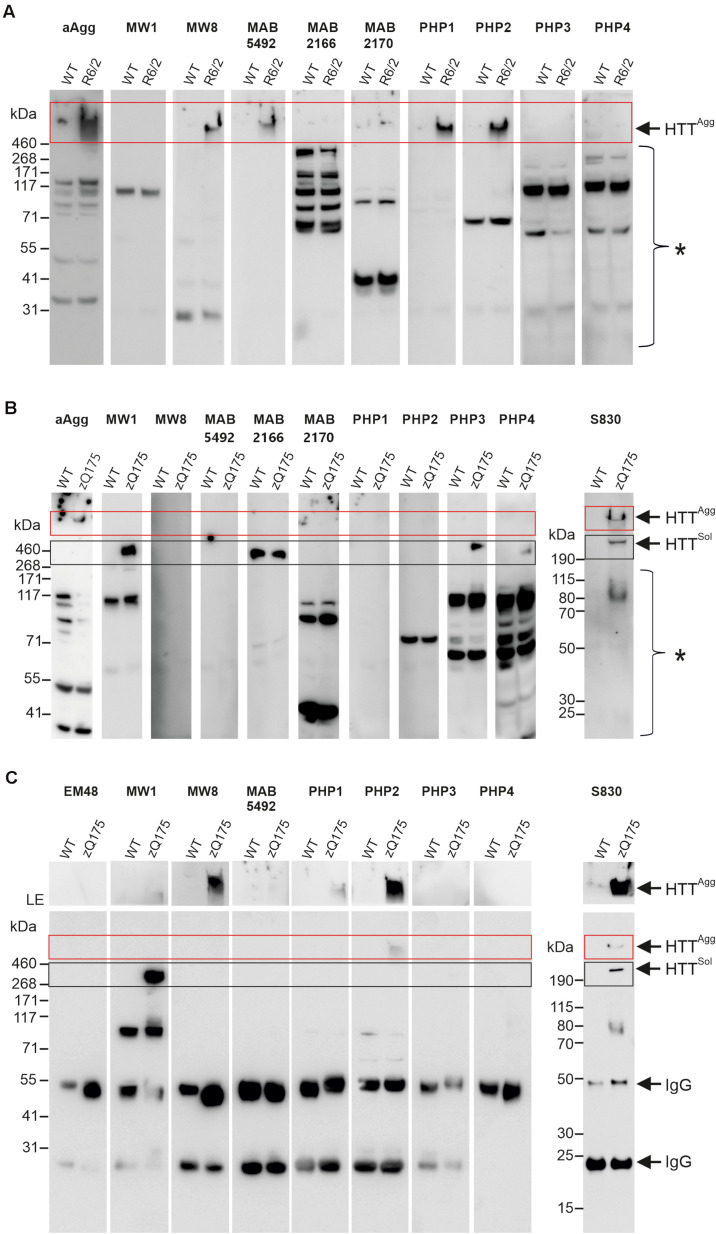
Analysis of mHTT aggregates and soluble protein in brain homogenates from R6/2 and zQ175 mice. **(A)** 50 μg of total brain homogenates prepared from 12-week-old R6/2 and wild-type (WT) mice were analyzed on a denaturing NuPage gel and by immunoblotting using the antibodies indicated above the lanes. For preparation of brain homogenates, BLB was used. HTT^Agg^ and a red box indicate mHTT aggregates in the gel pockets. A bracket with an asterisk indicates immunoreactive bands which cannot be assigned to specific proteins. **(B)** 100 μg of total brain homogenate prepared from 12-month-old zQ175 and wild-type (WT) mice was analyzed on a denaturing NuPage gel and by immunoblotting using the indicated antibodies. For preparation of brain homogenates RIPA buffer was used. HTT^Agg^ and a red box indicate mHTT aggregates in the gel pockets. HTT^sol^ indicates soluble ∼350 kDa HTT protein. A black box indicates soluble HTT, migrating at the expected size of ∼350 kDa. A bracket with an asterisk indicates immunoreactive bands which cannot be assigned to specific proteins. **(C)** Immunoprecipitations using different antibodies, as indicated on top of the gels; total brain homogenates (in BLB) derived from 12-month-old zQ175 and wild-type (WT) mice were applied. Immunoblots were developed with the respective antibodies used for immunoprecipitation and exposed for 20 s, with the exception of the S830 immunoblot, which was exposed for 30 s. The upper parts of blots were exposed separately for 5 min, to better visualize mHTT aggregates. LE, long exposure. HTT^Agg^ and a red box indicate mHTT aggregates in the gel pockets. HTT^sol^ and a black box indicate soluble FL HTT protein migrating at ∼350 kDa. IgG indicates heavy (∼50 kDa) and light (∼25 kDa) antibody chains.

Previous experiments demonstrated that seeding-competent mHTTex1 aggregates are SDS-stable, high molecular weight (HMW) structures readily detected in brains of transgenic R6/2 HD mice (Ast et al., 2018). In this HD model, a human mHTTex1 fragment with a pathogenic polyQ tract has a high propensity to form SDS-stable fibrillar protein aggregates (Scherzinger et al., 1999). Here, we first assessed whether a set of reported anti-HTT antibodies can detect mHTTex1 aggregates in crude mouse brain extracts of 12-week-old R6/2 mice by SDS-PAGE and immunoblotting. In R6/2 mouse brains, neuronal IBs are observed after 4 weeks with immunohistochemical methods (Landles et al., 2020). This suggests that HMW aggregates are also detectable by biochemical assays. We systematically tested 10 anti-HTT antibodies (Figure 2A, Supplementary Table 1 and Supplementary Figure 3) for their ability to detect SDS-stable aggregates in crude HD and control mouse brain extracts. Most of them were previously shown to recognize N-terminal mHTT fragments (Supplementary Table 1; Ko et al., 2001, 2018), suggesting that they are immunoreactive for mHTTex1 fragments that are formed in R6/2 HD brains. We found that five of the tested antibodies (aAgg, MW8, MAB5492, PHP1, and PHP2) indeed detect SDS-stable, HMW mHTTex1 aggregates in HD mouse brains, which are retained in the stacking gel (Figure 2A). It is important to note that with none of tested anti-HTT antibodies soluble mHTTex1 protein with a pathogenic polyQ tract was identified, indicating that this protein at an age of 12 weeks is exclusively present in its aggregated form in R6/2 brains.

Next, we assessed whether HMW, SDS-stable mHTT aggregates can also be detected in brains of zQ175 KI mice that express full-length mHTT. Crude brain extracts prepared from 12-month-old KI and wild-type control animals were systematically investigated by SDS-PAGE and immunoblotting using the same set of antibodies. In addition, the polyclonal antibody S830 was assessed. We could detect HMW mHTT aggregates with the antibodies aAgg and S830 (Figure 2B). However, aggregates were not detected with the antibodies MW8, MAB5492, PHP1, and PHP2, which showed immunoreactivity in R6/2 mouse brains (Figure 2A). This may be due to lower abundance of SDS-stable, HMW mHTT aggregates in zQ175 compared to R6/2 brains or to structural and morphological differences between mHTT aggregates in zQ175 and R6/2 mouse brains. However, we observed that the anti-HTT antibodies MW1, PHP3, and PHP4 recognize FL mHTT protein migrating at ∼350 kDa in SDS-gels (Figure 2B). This indicates that these antibodies preferentially recognize FL soluble mHTT but not the HMW aggregates retained in the stacking gel. Interestingly, both wild-type and mutant FL HTT were detected with the antibody MAB2166, which recognizes an epitope located downstream of the polyQ tract (Supplementary Figure 3). Only with the polyclonal antibody S830 both HMW aggregates and soluble FL mHTT were detected on immunoblots (Figure 2B). However, with none of the applied antibodies soluble truncated N-terminal mHTT fragments could be detected on immunoblots, suggesting that such fragments are only present in an aggregated form in zQ175 mouse brains. Together these biochemical experiments show that HMW, SDS-stable mHTT aggregates can be detected in brains of R6/2 and zQ175 KI mice under denaturing conditions by immunoblotting using different anti-HTT antibodies.

### Enrichment of mHTT Aggregates From zQ175 Mouse Brain Extracts by Immunoprecipitation

To investigate the morphology and seeding activity of mHTT aggregates formed in zQ175 mouse brains, we first established an antibody-based immunoprecipitation (IP) method using magnetic beads (Vila et al., 2010). We then systematically tested 8 different anti-HTT antibodies for their ability to IP mHTT aggregates under native conditions from crude brain extracts. Analysis of immunoprecipitates by SDS-PAGE and immunoblotting revealed that the anti-HTT antibodies MW8, PHP2 and S830 can enrich HMW, SDS-stable mHTT aggregates from zQ175 brain extracts (Figure 2C), while no or very low amounts of aggregates were immunoprecipitated with the antibodies MW1, MAB5492, PHP1, PHP3, and PHP4. Interestingly, we observed that the antibodies MW8 and PHP2 can IP mHTT aggregates from zQ175 mouse brain extracts, while they did not react with HMW, SDS-stable mHTT structures on immunoblots (Figure 2B). This suggests that these antibodies bind to mHTT aggregates under native conditions and can enrich such structures from crude mouse brain extracts. However, our investigations also indicate that the MW8 antibody, which recognizes a neoepitope that is only exposed at the C-terminus of HTTex1 fragments but not on FL HTT (Landles et al., 2010) can readily IP mHTT aggregates from mouse brain extracts (Figure 2C). This suggests that this antibody recognizes aggregates that are formed of mHTTex1 fragments. Such specific N-terminal mHTT fragments are known to be formed by alternative splicing in brains of HD KI mice and patient brains, which express FL mHTT (Sathasivam et al., 2013; Neueder et al., 2017). To assess whether MW8 can indeed immunoprecipitate mHTTex1 aggregates, we also performed immunoprecipitations with brain extracts prepared from R6/2 mice, which exclusively express a mHTTex1 fragment with a pathogenic polyQ tract but no FL mHTT protein (Mangiarini et al., 1996). We found that HMW, SDS-stable mHTTex1 aggregates can be immunoprecipitated from R6/2 mouse brain extracts but not from WT controls using the MW8 antibody (Supplementary Figure 4), confirming that this antibody recognizes *in vivo* aggregated mHTTex1 fragments under native conditions. It is important to note that neither from zQ175 nor from R6/2 brain extracts soluble mHTTex1 fragments could be enriched with MW8 IPs (Figure 2C and Supplementary Figure 4), suggesting that this N-terminal fragment is only present in its aggregated form in HD mouse brains.

Finally, we demonstrated that the polyclonal antibody S830 can enrich both insoluble aggregates and soluble mHTT from crude protein extracts, while the antibody MW1 exclusively precipitates soluble FL mHTT (Figure 2C).

### Antibody-Enriched mHTT Aggregates From zQ175 Mouse Brain Extracts Are Seeding-Competent, Fibrillar Structures

**FIGURE 3:**
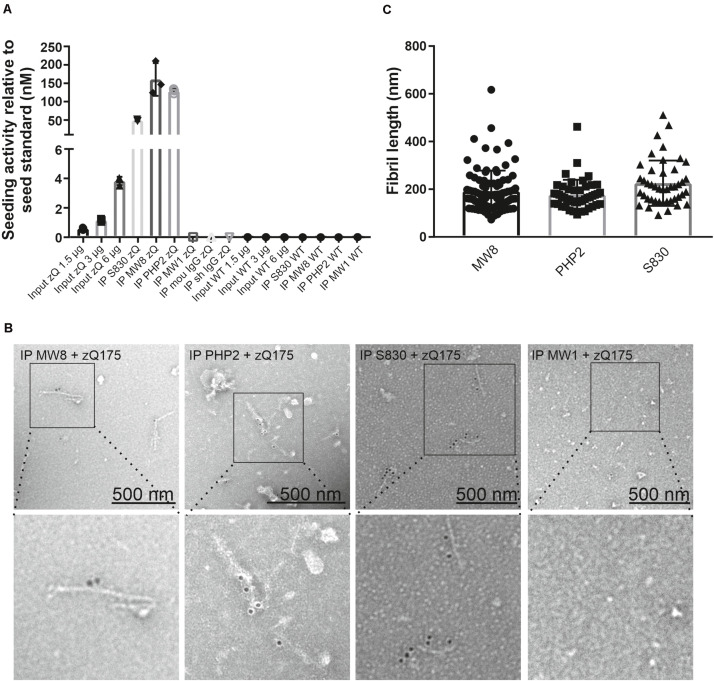
Quantification of mHTT seeding activity and detection of mHTT fibrils in immunoprecipitates enriched from zQ175 mouse brain homogenates. **(A)** Immunoprecipitates and total brain homogenates (input) prepared from WT and zQ175 (zQ) mice were analyzed by FRASE assay. Seeding activities were normalized to the seed standard. Data are mean ± SD of three technical replicates. **(B)** TEM analysis with aAgg-immunogold labeling of eluates derived from S830, PHP2, and MW8 immunoprecipitations of zQ175 mouse brain homogenates. As a control, an eluate from a MW1 immunoprecipitation experiment with a zQ175 brain homogenate is shown. Pictures in the bottom row show magnifications of areas indicated in pictures in the top row. Fibril length was determined with the iTEM software **(C)**. For the MW8 IP, *n* = 95, for the PHP2 IP, *n* = 48 and for the S830 IP, *n* = 44 fibrils were measured. Mean fibril length is 192 nm ± 87 for the MW8 IP, 177 nm ± 63 for the PHP2 IP and 226 nm ± 95 for the S830 IP.

To address the question of whether mHTT aggregates enriched from crude mouse brain extracts are seeding-competent, we added immunoprecipitates from zQ175 and WT control brains to FRASE assays. We found that the HMW, SDS-stable mHTT aggregates, which are immunoprecipitated with the antibodies S830, MW8 and PHP2 from zQ175 mouse brain extracts are highly seeding-competent structures (Figure 3A). In strong contrast, immunoprecipitated FL mHTT, which was enriched from zQ175 brain extracts with the MW1 antibody did not show seeding activity in FRASE assays, indicating that mHTT aggregates rather than the soluble FL protein stimulate the spontaneous aggregation of reporter proteins Ex1Q48-CyPet and -YPet in FRASE assays.

Next, we utilized immunoelectron microscopy (IEM) to investigate the morphology of antibody-enriched mHTT protein aggregates. We observed multiple mHTT fibrils in S830, PHP2 and MW8 immunoprecipitates but not in controls (Figure 3B), indicating that indeed fibrillar, structures are responsible for the observed seeding activity in FRASE assays (Figure 3A).

Finally, we quantified the lengths of the antibody-enriched mHTT fibrils, which were released from antibody beads by short-time treatment with an acidic buffer solution. We found that mHTT fibrils immunoprecipitated with the antibodies MW8, PHP2, and S830 on average have lengths of ∼192, ∼177, and ∼226 nm, respectively (Figure 3C), indicating that relatively small fibrillar mHTT aggregate species are enriched with different aggregate-specific HTT antibodies from 12-month-old zQ175 mouse brains. Together, these biochemical investigations strongly support our hypothesis that ordered, seeding-competent mHTT fibrils are present in brains of zQ175 KI mice.

### Soluble Fractions Prepared From zQ175 Mouse Brains Contain Seeding-Competent mHTT Structures

**FIGURE 4:**
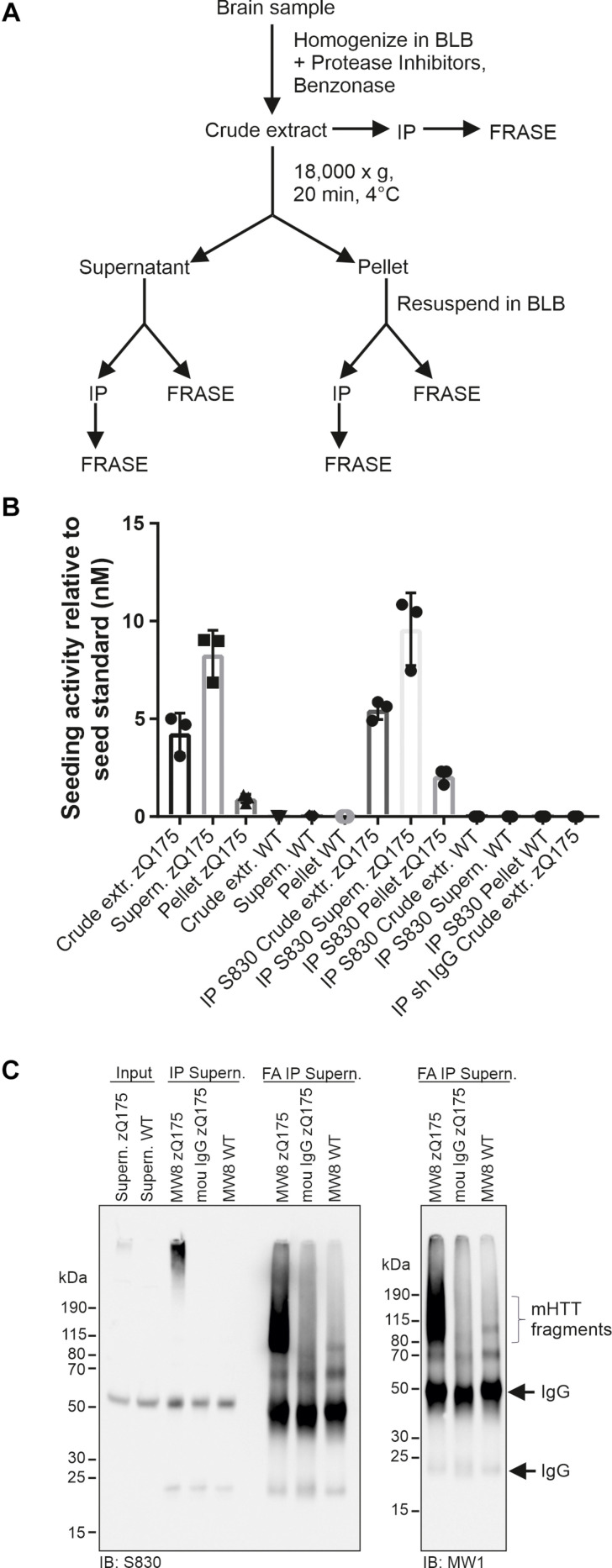
Analysis of HTT seeding activity in soluble and insoluble zQ175 brain fractions. **(A)** Schematic representation of fractionation and analysis steps. **(B)** Analysis of mHTT seeding activity using different samples derived from fractionations of zQ175 and WT mouse brain homogenates following panel **(A)**. Immunoprecipitations (IP) were performed with the antibody S830 and a control IgG. Seeding activity was normalized to the seed standard. Data are mean ± SD of three technical replicates. **(C)** IP was performed with supernatant fraction (shown in panel **(A)**) derived from WT and zQ175 mouse brain homogenates using antibody MW8 and mouse (mou) IgG. Immunoprecipitates were treated with formic acid (FA). Antibodies used for immunoblotting (IB) are indicated below the blots. Input, 200 μg supernatant of zQ175 and WT brain homogenate.

Previous investigations indicate that mHTT fibrils in cells accumulate as insoluble protein aggregates in IBs (Waelter et al., 2001; Bauerlein et al., 2017). However, evidence was also presented that they are soluble, diffusible structures (Sahoo et al., 2016; Ast et al., 2018) that potentially can spread from cell to cell (Iadanza et al., 2018). To assess whether soluble, seeding-competent mHTT structures are present in mouse brain extracts and can be enriched with antibodies, we performed a centrifugation experiment to separate soluble from insoluble proteins. Crude protein homogenates prepared from 12-month-old zQ175 and control mouse brains were centrifuged for 20 min at 18,000 × *g* and the resulting supernatant and pellet fractions were analyzed by FRASE (Figure 4A). We found highHSA in the supernatant fraction, indicating that soluble, seeding-competent mHTT fibrils are present in HD mouse brains. In strong contrast, such activity was not detected in the supernatant and pellet fractions prepared from wild-type control brains (Figure 4B).

Next, we performed immunoprecipitation experiments with both pellet and supernatant fractions using the S830 anti-HTT antibody. The precipitated material was analyzed for HSA in FRASE assays. We obtained a similar result as with the initial supernatant and pellet fractions (Figure 4B), supporting our hypothesis that soluble, seeding-competent mHTT aggregates are detectable in HD mouse brains.

Finally, we assessed whether the seeding-competent mHTT structures present in supernatant fractions consist of truncated N-terminal fragments. To address this question mHTT aggregates were first precipitated from the soluble fraction using the MW8 antibody and subsequently treated with formic acid (FA), which was previously shown to promote the dissociation of amyloidogenic mHTT fibrils into monomers (Hazeki et al., 2000). Samples were finally analyzed by SDS-PAGE and immunoblotting using the antibodies S830 and MW1. We observed that upon FA treatment a smear of S830- and MW1-reactive mHTT fragments (∼80–190 kDa) was detectable (Figure 4C), indicating that the HMW mHTT assemblies present in the soluble fraction predominantly consist of truncated N-terminal fragments. Together, these studies indicate that small seeding-competent mHTT aggregates consisting of N-terminal fragments are indeed present in soluble protein factions in brains of zQ175 KI mice.

### The Human Antibodies Ab-A and Ab-B Immunoprecipitate Seeding-Competent, Fibrillar mHTT Aggregate Species From HD Mouse Brains

**FIGURE 5:**
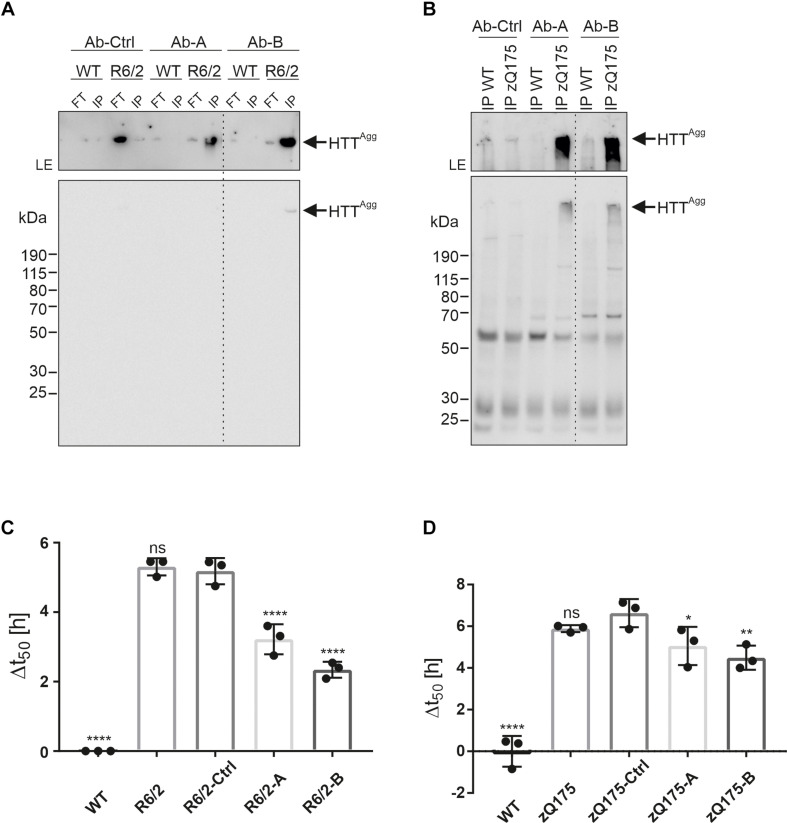
Enrichment of mHTT aggregates from mouse brain extracts using human anti-HTT antibodies. **(A)** The human HTT antibodies Ab-A and Ab-B and an isotype control (Ab-Ctrl) were used for immunoprecipitation of mHTT aggregates from WT and R6/2 brain homogenates. Immunoprecipitates (IP) and flow-through samples (FT) were analyzed by immunoblotting on a denaturing gel using MW8 antibody. The western blot was exposed for 5 s. To better visualize the aggregates in the gel pockets, the upper part of the blot was exposed separately for 5 min. LE, longer exposure. HTT^Agg^ indicates mHTTex1 aggregates. **(B)** Antibodies Ab-A, -B, and -Ctrl were used for immunoprecipitation of mHTT aggregates from WT and zQ175 mouse brain homogenates. Immunoprecipitates (IP) were analyzed by immunoblotting on a denaturing gel using the PHP2 antibody. The western blot was exposed for 10 s. The upper part of the blot was exposed separately for 1 min. LE, longer exposure **(C)** Analysis of WT and R6/2 brain homogenates, and FT samples derived from immunoprecipitations using Ab-A (R6/2-A), Ab-B (R6/2-B), and Ab-Ctrl (R6/2-Ctrl) in the FRASE assay. Δt50 is displayed as mean ± SD of four technical replicates. Statistical analysis: One-way ANOVA followed by Dunnett’s multiple-comparisons test reveals a significant difference in Δt50 values between WT and R6/2-Ctrl (****), R6/2-A and R6/2-Ctrl (****), and R6/2-B and R6/2-Ctrl (****) samples. ****, adjusted (adj.) *p* value = 0.0001. **(D)** Analysis of WT and zQ175 brain homogenates, and FT samples derived from immunoprecipitations using Ab-Ctrl (zQ175-Ctrl), Ab-A (zQ175-A), and Ab-B (zQ175-B) in the FRASE assay. Δt50 is displayed as mean ± SD of four technical replicates. Statistical analysis: One-way ANOVA followed by Dunnett’s multiple-comparisons test reveals a significant difference in Δt50 valuesbetween WT and zQ175-Ctrl (****, adj. *p* value = 0.0001), zQ175-A and zQ175-Ctrl (*, adj. *p* value = 0.049) and zQ175-B and zQ175-Ctrl (**, adj. *p* value = 0.0091) samples.

By screening human memory B cell repertoires of healthy elderly subjects and HD patients, we generated recombinant antibodies that preferentially bind to aggregated recombinant mHTTex1 fragments with a pathogenic polyQ tract (Sevigny et al., 2016; Maier et al., 2018). Two high-affinity human-derived anti-HTT antibodies (Ab-A and Ab-B) and a human isotype control antibody (Ab-Ctrl) were analyzed for their ability to immunoprecipitate mHTT aggregates under native conditions from crude brain extracts from 12-week-old R6/2 and 9-month-old zQ175 HD mice. Analysis of immunoprecipitates by SDS-PAGE and immunoblotting revealed that both anti-HTT antibodies Ab-A and Ab-B precipitated HMW SDS-stable mHTT aggregates from R6/2 (Figure 5A) and zQ175 mouse brain homogenates (Figure 5B), while no or only minimal amounts of aggregates were detectable after immunoprecipitation with the human isotype control. Electron microscopy analysis of immunoprecipitates revealed the presence of fibrillar structures following precipitation with Ab-A and -B but not the isotype control antibody (Supplementary Figure 5).

To address the question whether human anti-HTT antibodies can bind and deplete seeding-competent mHTT aggregates from HD mouse brain homogenates, we quantified HSA in crude protein extracts after immunodepletion. We found that HSA was significantly decreased after treatment with the anti-HTT antibodies A and B but not the isotype control (Figures 5C,D). Thus, both human mHTT aggregate-specific antibodies are capable of removing fibrillar, seeding-competent mHTT aggregates from crude brain homogenates of R6/2 and zQ175 HD mice.

## Discussion

Several lines of experimental evidence indicate that the formation of mHTT aggregates is associated with the appearance of behavioral symptoms in HD mouse models (Li et al., 2001; Ast et al., 2018). Whether aggregate assembly plays a causative role in the pathogenic process or rather is an epiphenomenon that does not significantly contribute to disease development (Slow et al., 2005), however, is still a matter of debate.

Here, we systematically applied a FRASE assay, immunoprecipitations and high-resolution imaging methods to investigate the formation of fibrillar mHTT protein aggregates in brains of heterozygous zQ175 KI HD mice, which endogenously express FL mHTT with a pathogenic polyQ tract (Menalled et al., 2012). We measured high HSA in crude brain protein extracts prepared from 6-month-old zQ175 KI mice, indicating that seeding-competent mHTT fibrils are a prominent aggregate species in this HD model.

Previous investigations with HD transgenic mice and cell models have demonstrated that neuronal IBs are densely packed with fibrillar mHTT aggregates (Davies et al., 1997; Waelter et al., 2001; Bauerlein et al., 2017). This might suggest that mHTT fibrils in neurons are predominantly insoluble structures that either accumulate at specific subcellular locations by nucleation (Wagner et al., 2018) or are actively transported to IBs for deposition (Kopito, 2000). The local concentration of insoluble aggregated mHTT fibrils in IBs may be an important coping mechanism to protect neurons from aggregate toxicity (Arrasate et al., 2004). Our investigations of mouse brain extract with FRASE assays, however, suggest that a large fraction of the mHTT fibrils formed in HD mouse brains is soluble rather than insoluble (Figure 4B). The relatively small ∼200 nm long mHTT fibrils observed by IEM (Figure 3B) are likely to be diffusible structures that can move from one place to another in the cytoplasm and have the potential to perturb multiple biological functions such as axonal transport (Lee et al., 2004) or the release of neurotransmitters in synapses (Li et al., 2001). Our observation that mHTT fibrils in neurons are largely small soluble structures (Figure 4B) is supported by previous investigations showing that mHTTex1 fibrils accumulate rapidly as insoluble aggregates in large spherical IBs but at the same time are also present in a soluble form in the cytoplasm of mammalian cells (Sahl et al., 2016). It is important to note that IBs are very complex protein condensates that contain many other cellular proteins besides insoluble mHTTex1 fibrils, as e.g., chaperones, ubiquitin or different components of the ubiquitin proteasome system (Waelter et al., 2001; Hosp et al., 2017). The composition of small, soluble mHTT fibrils in HD models and patient brains still needs to be investigated in depth with mass spectrometry-based methods.

The detection of seeding-competent mHTT fibrils in brains of zQ175 mice with immunoprecipitations and IEM (Figure 3B) is not unexpected. Previously, fibrillar mHTT aggregates were successfully enriched from brains of R6/2 and *Hdh*Q150 KI mice using a Seprion ligand (Sathasivam et al., 2010) that binds large, amyloidogenic protein assemblies, soluble oligomers and protofibrils. Similarly, small ordered mHTTex1 aggregates with a typical fibrillar morphology were isolated from HD mouse brain extracts using biochemical methods (Diaz-Hernandez et al., 2004; Ast et al., 2018). Finally, utilizing IEM methods, it was demonstrated that neuropil EM48-reactive inclusions in brains of HD KI mice contain fibrillar N-terminal mHTT assemblies (Li et al., 2001). Strikingly, such aggregates are formed early in the disease process in HD-relevant brain regions such as the striatum and are associated with axonal degeneration, suggesting that they play a critical role in pathogenesis. We propose here that the antibody-enriched mHTT fibrils from brain extracts of zQ175 mice might predominantly originate from the cytoplasm, which likely contains high amounts of mHTT aggregates (Maat-Schieman et al., 1999; Cicchetti et al., 2014). However, it may well be that small, seeding-competent mHTT fibrils formed in neuronal nuclei are also enriched with the mHTT aggregate-specific antibodies. Further studies are necessary to assess the relative abundance of mHTT aggregate species in different subcellular compartments and their seeding activity.

Through immunoprecipitations followed by IEM experiments, we could show that antibodies such as MW8 and PHP2 are able to enrich seeding-competent, fibrillar mHTT aggregates from mouse brain extracts (Figures 3A,B). In strong contrast, FL mHTT migrating at ∼350 kDa was specifically enriched from brain extracts utilizing the MW1 antibody. These results are in agreement with previous studies indicating that aggregated and non-aggregated forms of mHTT expose distinct epitopes and therefore require different anti-HTT antibodies to be detected. While the pathogenic polyglutamine (polyQ) tract in FL mHTT is exposed on the protein surface and can be recognized by the polyQ-specific MW1 antibody, it is not exposed in β-sheet-rich fibrillar mHTT aggregates (Wagner et al., 2018). Thus, such structures cannot be immunoprecipitated with the MW1 antibody. Other domains such an α-helical proline-rich domain (PRD) are predominantly exposed in fibrillar mHTT aggregates, which can be recognized by aggregate-specific mHTT antibodies such as PHP2 (Ko et al., 2018).

Interestingly, fibrillar mHTT aggregates could be enriched from zQ175 brain extracts using the monoclonal antibody MW8 (Figure 3B). This conformation-sensitive antibody was previously shown to specifically recognize the C-terminus of HTTex1 fragments, which is not present in longer N-terminal HTT fragments or the FL protein (Landles et al., 2010). Thus, the MW8 antibody possibly enriches structures from mouse brain extracts that contain mHTTex1 fragments. Our results are therefore consistent with previous observations indicating that mHTTex1 fragments are produced in brains of zQ175 mice through abnormal splicing (Sathasivam et al., 2013). These peptides are highly aggregation-prone; they may function as seeds and drive the co-aggregation of longer N-terminal HTT fragments with pathogenic and non-pathogenic polyQ tracts, likely formed in HD brains by proteolytic cleavage of FL HTT (Ratovitski et al., 2009). Cell-free aggregation studies with recombinant proteins have shown that N-terminal HTT fragments with short and long polyQ tracts indeed can co-assemble into stable mixed fibrils (Busch et al., 2003), suggesting that polymorphic mHTT fibrils with distinct biological activities may also form *in vivo*. Furthermore, it is likely that at least a fraction of the mHTT fibrils formed in neurons undergoes post-translational modification, e.g., through the attachment of ubiquitin chains, which additionally enhances their polymorphic nature (Juenemann et al., 2015).

MW8-based ELISAs and homogenous time-resolved fluorescence (HTRF) assays were recently applied successfully to detect mHTT aggregates in zQ175 mouse brain extracts (Reindl et al., 2019). However, this approach does not provide information about the morphology and structure of the mHTT aggregates. Our investigations suggest that seeding-competent mHTT aggregate species indeed are detected with MW8 in ELISAs. However, it is important to note that current antibody-based mHTT aggregate detection assays (Reindl et al., 2019; Landles et al., 2021), in strong contrast to the FRASE method applied here, might also detect non-fibrillar mHTT assemblies in tissue samples. Such assemblies were previously described in different HD model systems (Sathasivam et al., 2010) and are thought to play a key role in pathogenesis (Hoffner and Djian, 2014). Similar to small mHTT fibrils, they are highly mobile structures. A soluble pool of MW1-immunoreactive mHTT oligomers formed of N-terminal fragments was previously identified in brains of HdhQ150 KI mice using the SEC-FRET technology (Marcellin et al., 2012). Such structures are less stable than β-sheet-rich mHTT fibrils. PolyQ tracts, readily recognized by the MW1 antibody, are exposed on their surface. Via their polyQ sequences, these dynamic mHTT structures may interact with other cellular proteins and perturb their cellular functions (Kwon et al., 2018). However, we suggest that both fibrillar and non-fibrillar mHTT structures are formed in neurons of zQ175 KI mice and contribute independently to the pathogenic process.

Together our studies show that small, seeding-competent mHTT fibrils are prominent aggregate species in brains of zQ175 HD mice. We suggest that they have the potential of driving the pathogenic process in zQ175 HD KI mice and patients, for these reasons: They are detected in brains of very young mice; they appear before the onset of symptoms; finally, their abundance increases progressively with disease development in typically affected brain regions such as the striatum.

## Materials and Methods

### Antibodies

Most monoclonal antibodies used in this study were obtained by commercial suppliers (see Supplementary Table 1). Production and characterization of the antibodies aAgg (Scherzinger et al., 1999), PHP1-4 (Ko et al., 2018), and S830 (Neueder et al., 2017) were described previously. The concentrations of antibodies used for immunoprecipitations, immunoblots, and immuno-EM studies are described in the methods section below. Secondary colloidal gold-labeled anti-rabbit antibodies were used for transmission electron microscopy (TEM) studies, while peroxidase or HRP-labeled secondary antibodies were used for western blotting. For immunoprecipitations mouse IgG1 (Thermo Scientific) or sheep IgG (Millipore) were utilized as controls. Human anti-HTT antibodies A and B were derived from a de-identified blood lymphocyte library collected from healthy elderly subjects or HD patients utilizing previously reported procedures (Maier et al., 2018). High-affinity anti-HTT antibodies Ab-A and Ab-B were selected based on their preferential binding to aggregated recombinant human mHTTex1 fragments with a pathogenic polyQ49 tract compared to HTTex1 fragments with a normal polyQ21 tract using dot-blot and filter-retardation assays (Wagner et al., 2018).

### Mouse Breeding and Maintenance

All procedures were performed in accordance with the Animals (Scientific Procedures) Act 1986, complied with ARRIVE guidelines and were approved by the University College London Ethical Review Process Committee. zQ175 knock-in mice were generated by replacing exon 1 of mouse *Htt* with exon 1 from human *HTT*, carrying a highly expanded CAG repeat (Menalled et al., 2012), from which the neo-selectable marker had been removed (delta neo) (Franich et al., 2019). Mice were either bred in-house by backcrossing males to C57Bl/6J females (Charles River) or obtained from the CHDI Foundation colony at the Jackson Laboratory (Bar Harbor, Maine) on a C57Bl/6J background. R6/2 mice (Mangiarini et al., 1996) were bred by backcrossing R6/2 males to C57BL/6JOlaHsd × CBA/CaOlaHsd F1 females (B6CBAF1/OlaHsd, Envigo, Netherlands).

Within each colony, genetically modified and wild-type mice were group housed with up to five mice per cage, dependent on gender, but genotypes were mixed. Mice were housed in individually ventilated cages with Aspen Chips 4 Premium bedding (Datesand) and with environmental enrichment, which included chew sticks and a play tunnel (Datesand). They had unrestricted access to food (Teklad global 18% protein diet, Envigo) and water. The temperature was regulated at 21°C ± 1°C and animals were kept on a 12 h light-dark cycle. The animal facility was barrier-maintained and quarterly non-sacrificial FELASA screens found no evidence of pathogens.

R6/2 and zQ175 mice were genotyped and CAG repeat sizing was performed as previously described (Landles et al., 2021). The mean CAG repeat size ± SD was 190.23 ± 4.53 for 6-month-old zQ175, 194.45 ± 3.15 for 12-month-old zQ175, and 181.0 ± 4.0 for 12-week-old R6/2 mice.

### Protein Expression

*Escherichia coli* BL21-RP carrying pGEX-6P1_Ex1Q48, pGEX-6P1_Ex1Q48-CyPet, and pGEX-6P1_Ex1Q48-YPet were grown in 20 ml of LB medium containing 150 μg/ml ampicillin and 42 μg/ml chloramphenicol at 37°C and 210 rpm shaking overnight. On the next day, the culture was inoculated in 1 l of LB media and grown to an OD_600_ of 0.6. Then, expression was induced with 1 mM IPTG for 4 h. Cultures of induced bacteria were centrifuged at 3,501 × *g* for 20 min, and the resulting pellets were stored at −80°C. Cells were thawed on ice and resuspended in 20 ml of cold buffer I (50 mM NaH_2_PO_4_, 5 mM Tris, 150 mM NaCl, 1 mM EDTA, pH 8.0) containing 1 mg/ml lysozyme and protease inhibitors. After incubation for 30 min on ice, cells were sonicated on ice with eight 10 s bursts (45 s break between bursts) using a Branson sonicator 450 (output control 4, duty cycle constant). Then, TritonX-100 to a final concentration of 1% was added and incubation continued for 5 min on ice. The resulting lysate was clarified by centrifugation for 40 min at 26,892 g. Cleared lysates were incubated on a rotating wheel for 1 h at 4°C with 4 ml of 1:1 slurry of glutathione-agarose beads (Sigma-Aldrich) that had been washed three times and resuspended in buffer I. The beads were poured into a small polypropylene column, washed with 10 ml buffer I containing 0.1% Triton-X and protease inhibitors, followed by a wash with 10 ml buffer I without additives. The bound fusion protein was eluted with 4 ml of 20 mM glutathione (reduced) in buffer I (pH 8.6) and then dialyzed overnight in a 10 kDa MWCO membrane and buffer containing 50 mM Tris, 150 mM NaCl, 1 mM EDTA and 5% glycerol (pH 7.4).

### Western Blot Analysis

Protein extracts or magnetic protein G beads with immunoprecipitated HTT species were boiled with NuPAGE 4× sample buffer for 5 min and then loaded onto NuPAGE Novex 4–12% Bis-Tris gels (Thermo-Fisher Scientific, Waltham, MA, United States). Electrophoresis was performed according to a standard protocol followed by transfer of proteins onto a nitrocellulose membrane (0.45 μm; GE Healthcare Life Sciences, Munich, Germany) using a wet blotting system (Bio-Rad, Munich, Germany). The generated blots were incubated with primary antibodies (1:1000) and HRP-conjugated secondary antibodies (1:2000, Sigma-Aldrich, Taufkirchen, Germany). Immunoreactive proteins on membranes were visualized with WesternBright Quantum chemiluminescence substrate (Advansta, Menlo Park, CA, United States).

### Coomassie Staining

Protein extracts were boiled with NuPAGE 4× sample buffer for 5 min and then loaded onto NuPAGE Novex 4–12% Bis-Tris gels (Thermo Fisher Scientific, Waltham, MA, United States). After electrophoresis, gels were stained with Coomassie blue R (50% methanol, 9.2% acetic acid, 0.175 brilliant blue) overnight followed by destaining with water.

### FRASE Assay and Quantification of mHTT Seeding Activity

The FRASE assay was performed as described in Ast et al. (2018). To obtain the t_50_ values, the aggregation kinetics were curve fitted by Richard’s five-parameter dose-response curve using GraphPad Prism. To quantify seeding activity of mHTT aggregate species in tissue samples or IP eluates, a seed standard was employed. Delta t_50_ values of the different seed standard concentrations were used to generate an exponential function in MS Excel. Using this function, the seeding activity of samples relative to the seed standard (in nM) was calculated.

### Preparation of Seed Standard

Mutant huntingtin exon-1 aggregates (Ex1Q48) were prepared as described in Ast et al. (2018). Aggregates were sonicated on ice with six 10 s bursts (30 s break between bursts) using a BRANSON Sonifier 450 (output control 1, duty cycle 90%). Then, the sonicated aggregates (seeds) were diluted to concentrations of 100, 10, 1, 0.1, 0.01, and 0.001 nM, aliquoted and stored at −80°C until use.

### Thioflavin T Binding Assay

Ex1Q48 aggregates were mixed with 20 μM Thioflavin T (ThT) in 1× PBS in a volume of 25 μl. Fluorescence measurements of samples were performed in a plate reader (Infinite M200, Tecan, Männedorf, Switzerland) at an excitation wavelength of 420 nm and an emission wavelength of 485 nm (LeVine, 1999).

### Preparation of Tissue Homogenates

Frozen tissue (400–600 mg) was homogenized in 1 ml ice-cold brain lysis buffer (BLB: 10 mM Tris, 1 mM EDTA, 0.8 M NaCl, 10% Sucrose, pH 7.4, EDTA-free protease inhibitors) using a Precellys homogeniser (5,000 rpm, 2 × 20 s, break: 15 s). Then, the buffer volume was adjusted to 10-fold of volume, Benzonase (0.25 U/μl) was added and the homogenate incubated for 1 h at 4°C. Protein concentration was determined with the Pierce BCA assay (Thermo Fisher Scientific, Waltham, MA, United States) using BSA as a standard. Homogenates were aliquoted and stored at −80°C until further use. For FRASE assays, samples in BLB buffer were used.

For preparation of mouse brain homogenates in RIPA buffer, 400–600 mg brain was homogenized in 1 ml ice-cold 50 mM Tris pH 7.5 buffer using the Precellys homogenizer (5000 rpm, 2×20 s, break: 15 s). Then, buffer volume was adjusted to 10-fold of volume using 50 mM Tris pH 7.5, 150 mM NaCl, 1% Triton, 0.1% SDS, 0.5% Sodium deoxycholate, EDTA-free protease inhibitors and Benzonase (0.25 U/μl). Homogenates were incubated for 1 h at 4°C. Protein concentration was determined using the Pierce BCA assay (Thermo Fisher Scientific, Waltham, MA, United States). Homogenates were aliquoted and stored at −80°C until further use.

### Immunoprecipitations and Formic Acid Treatment

Huntingtin protein species were enriched from mouse brain homogenates through immunoprecipitation with magnetic protein G beads (Thermo Fisher Scientific, Waltham, MA, United States) coated with the respective antibodies. For IP experiments shown in Figure 2, 2 μl antibody was bound to 0.75 mg beads by incubation for 30 min at room temperature on a rotating wheel. Antibody-bound beads were washed three times in PBS and then incubated with 1.25 mg RIPA homogenate at 4°C overnight on a rotating wheel. After washing the beads three times in a buffer containing 50 mM Tris pH 7.5, 150 mM NaCl, 0.01% SDS, 0.05% sodium deoxycholate, 0.1% Triton X-100 and protease inhibitors, HTT protein species were released from the beads by denaturation (5 min, 95°C) in NuPage sample buffer. All other immunoprecipitations were performed with tissue homogenates prepared in BLB. Antibodies (0.5–1 μg) were bound to 0.375 mg beads as described above. After washing the beads, brain homogenates in BLB buffer were added and incubated at 4°C overnight. Unbound material was taken off (=Flow Through) and beads were washed three times in BLB. HTT protein species were released from the beads either by denaturation in NuPage sample buffer or by incubation in 12.5 μl elution buffer (100 M Glycine-HCl, pH 2.8) for 30 min at 4°C. Eluates were neutralized by addition of 1.2 μl 1 M Tris pH 10.

For formic acid treatment, after the last washing step in BLB, beads with immunoprecipitates were resuspended in 100 μl 98% formic acid and incubated for 1 h at 37°C with 300 rpm shaking. Then, the bead formic acid solution was put to a magnet and the supernatant transferred to a new tube. After evaporation of the supernatant, elution buffer and 1 M Tris pH 10 were added and pipetted several times up and down, to solubilize proteins.

### Transmission Electron Microscopy (TEM)

For TEM investigations, samples (5 μl IP eluate) were adsorbed onto formvar-carbon coated grids (Plano) by 10 min incubation at room temperature. After two times washing in 1× PBS containing 1% BSA and 0.12% glycine (wash buffer) for 5 min, samples on grids were incubated with aAgg antibody, diluted 1:50 in wash buffer for 15 min at room temperature.

Samples were washed again and incubated with immunogold-labeled goat anti-rabbit antibody (S830 IP: 18 nm Colloidal Gold AffiniPure Goat Anti-Rabbit IgG; PHP2 and MW8 IP: 12 nm Colloidal Gold AffiniPure Goat Anti-Rabbit IgG), diluted 1:30 in wash buffer, for 10 min. Grids were washed two times in 1× PBS and five times in deionized water, before they were stained with 5% uranyl acetate, and analyzed using the Zeiss EM 910 microscope. Pictures were taken and analyzed with the iTEM software (EMSIS GmbH, Münster, Germany).

## Data Availability Statement

The original contributions presented in the study are included in the article/Supplementary Material, further inquiries can be directed to the corresponding author/s.

## Ethics Statement

The animal study was reviewed and approved by University College London Ethical Review Process Committee.

## Author Contributions

FS, NP, NN, and AB planned and performed the immunoprecipitations, FRASE assays, TEM and biochemical analysis. SK performed TEM analysis. AK generated PHP1-4 antibodies. GB discussed the experiments and results. MM, JG, and JP provided and contributed to the generation and characterization of human antibodies Ab-A, -B, and -Ctrl antibodies. MAM, BT, and GB provided mouse tissues. FS, SS, AB, and EW co-ordinated the study and edited the manuscript. EW designed the study and wrote the manuscript, with contributions from JP, GB, MM, SS, and AB. All authors contributed to the article and approved the submitted version.

## Conflict of Interest

MM and JG are co-inventors on patent number WO2016016278A2 entitled “Human-derived anti-huntingtin (htt) antibodies and uses thereof.” MM and JG are employees and shareholders of Neurimmune AG, Switzerland. The remaining authors declare that the research was conducted in the absence of any commercial or financial relationships that could be construed as a potential conflict of interest.
