# Supplementary figures and images for: Small, Seeding-Competent Huntingtin Fibrils Are Prominent Aggregate Species in Brains of zQ175 Huntington’s Disease Knock-in Mice
Source: Front Neurosci. 2021 Jun 22;15:682172. doi: 10.3389/fnins.2021.682172 (PMC8257939; doi:10.3389/fnins.2021.682172)

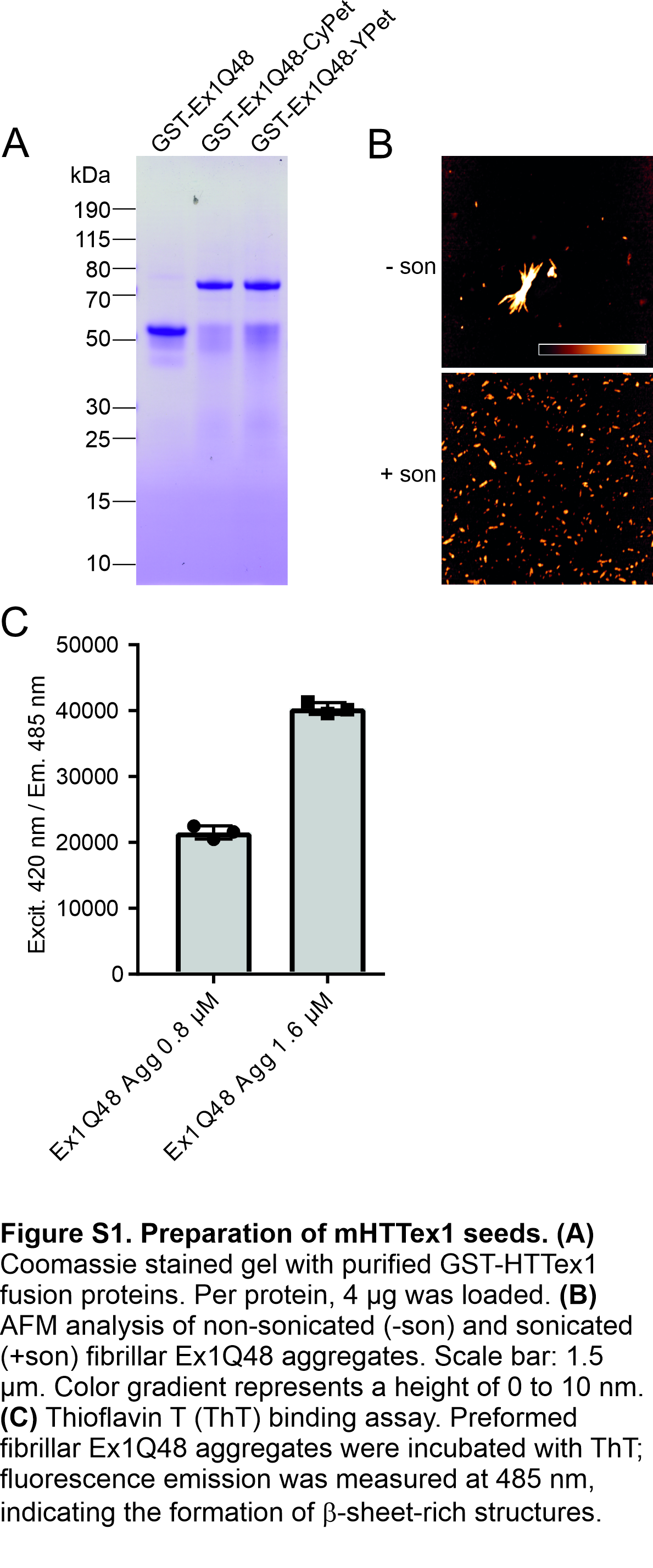

Supplement: Supplementary file 1 [file Image_1.TIF]

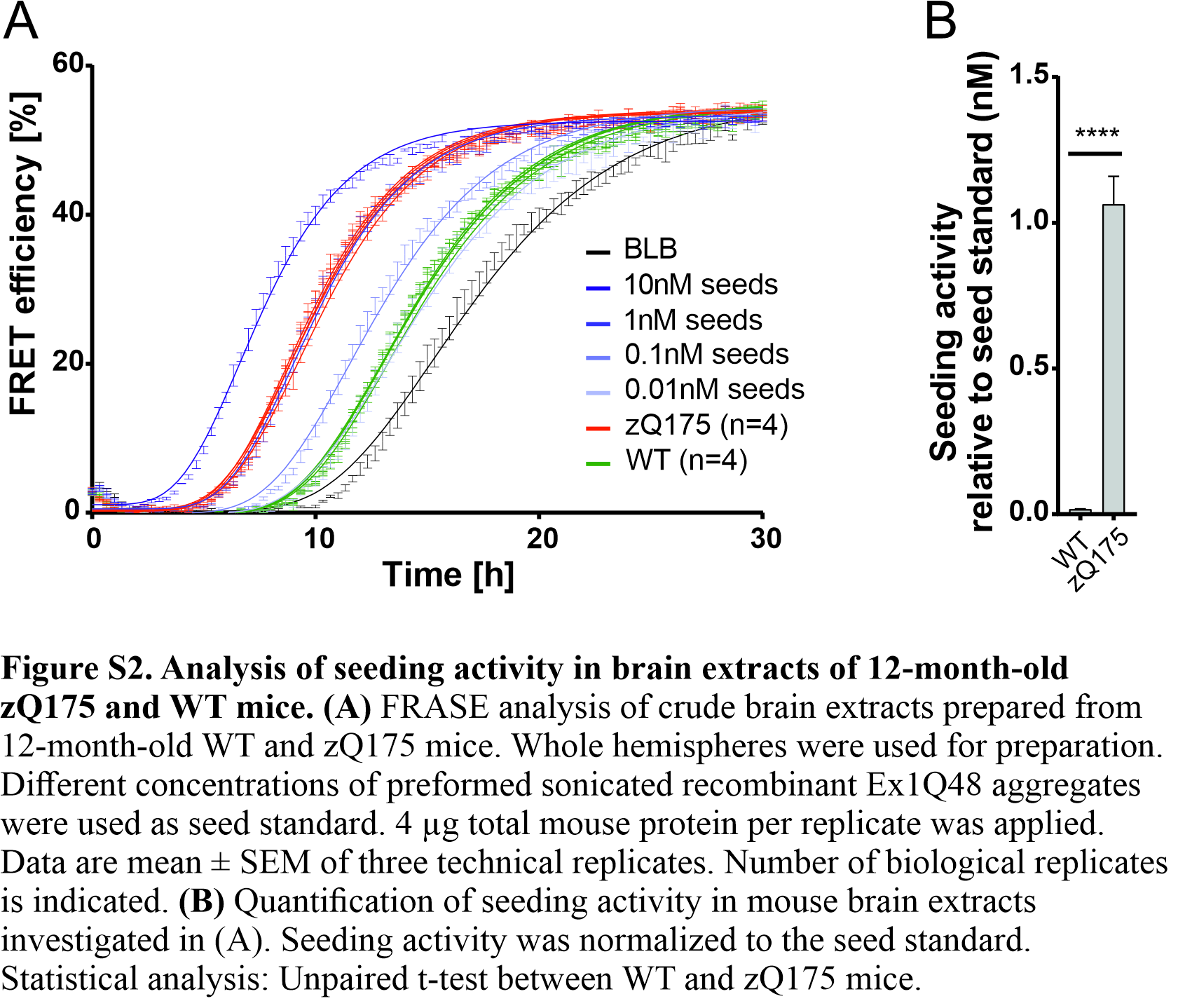

Supplement: Supplementary file 2 [file Image_2.TIF]

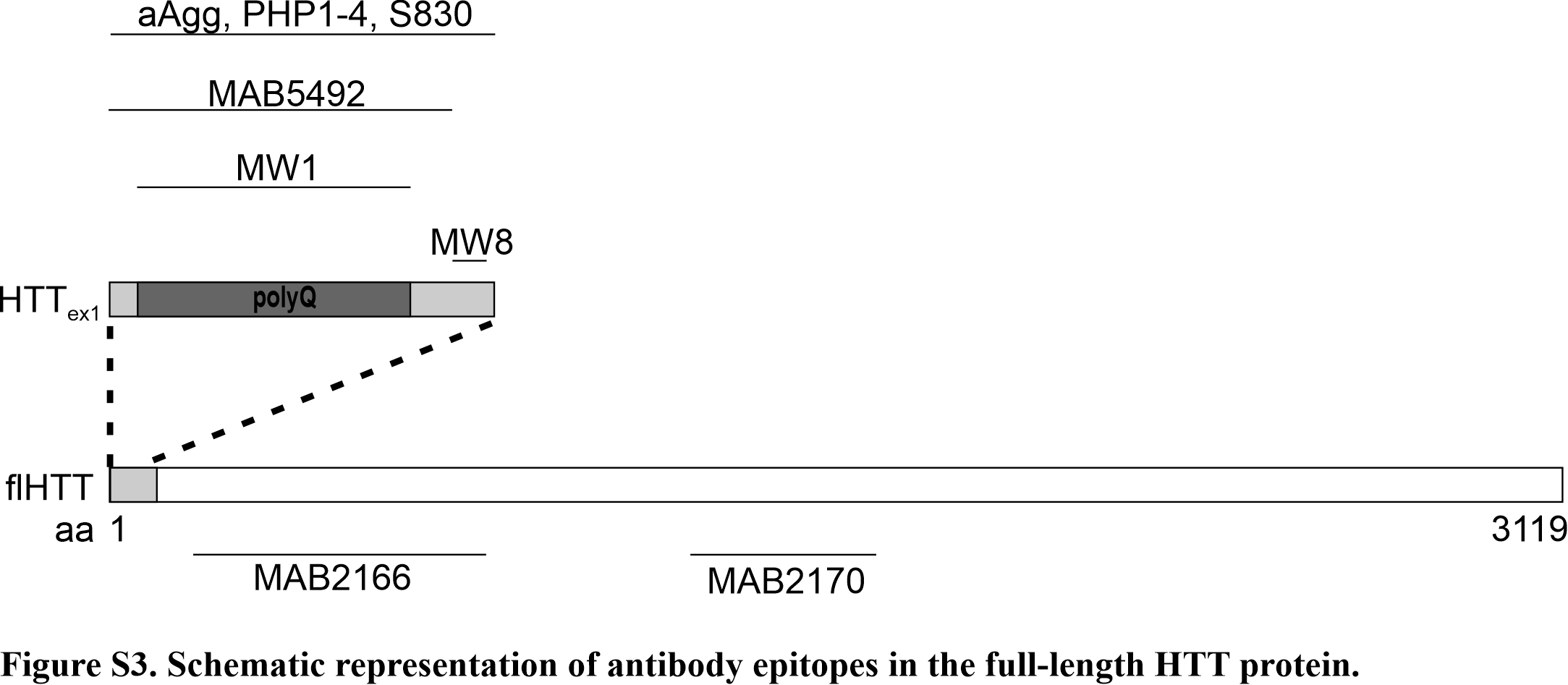

Supplement: Supplementary file 3 [file Image_3.TIF]

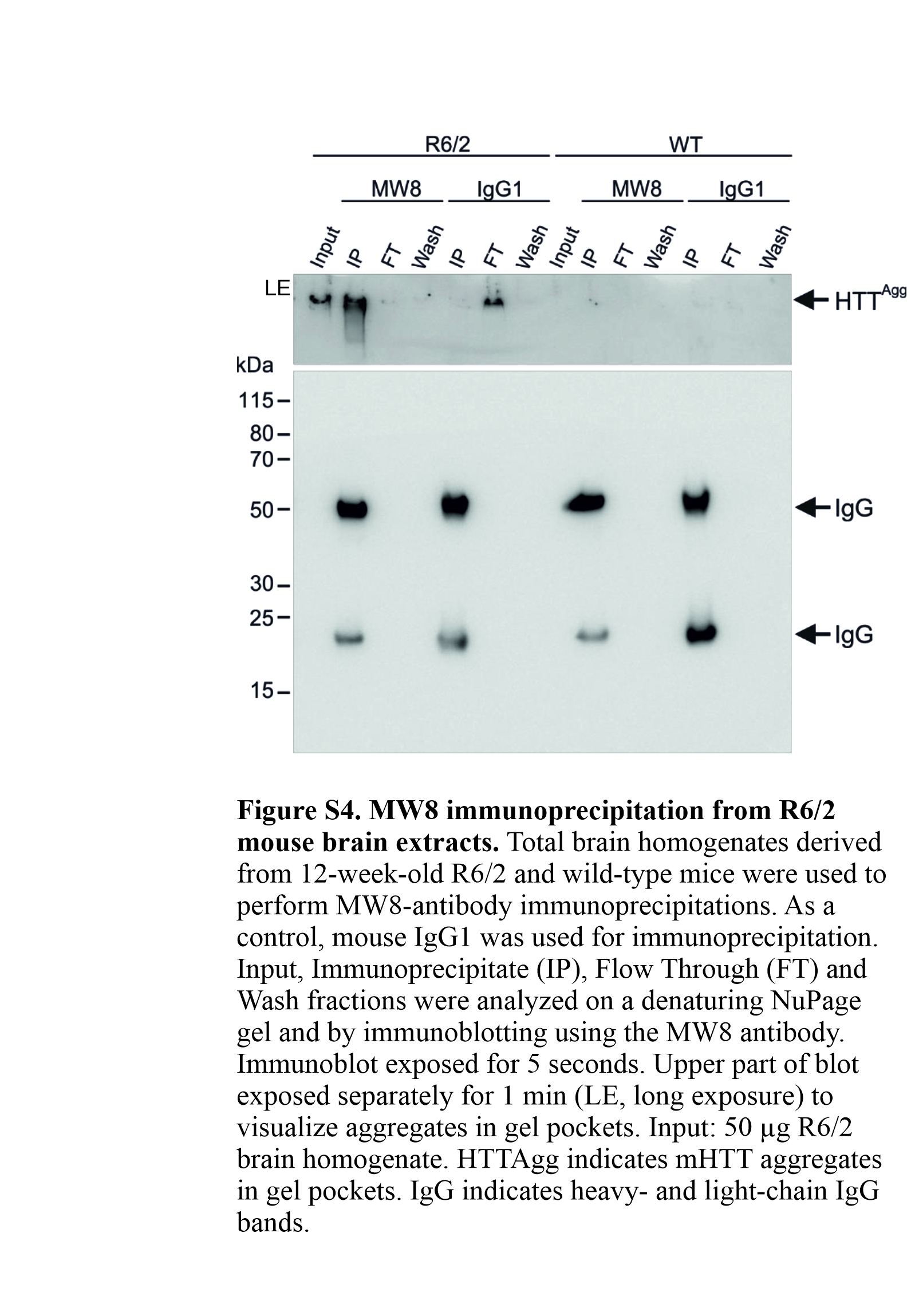

Supplement: Supplementary file 4 [file Image_4.TIF]

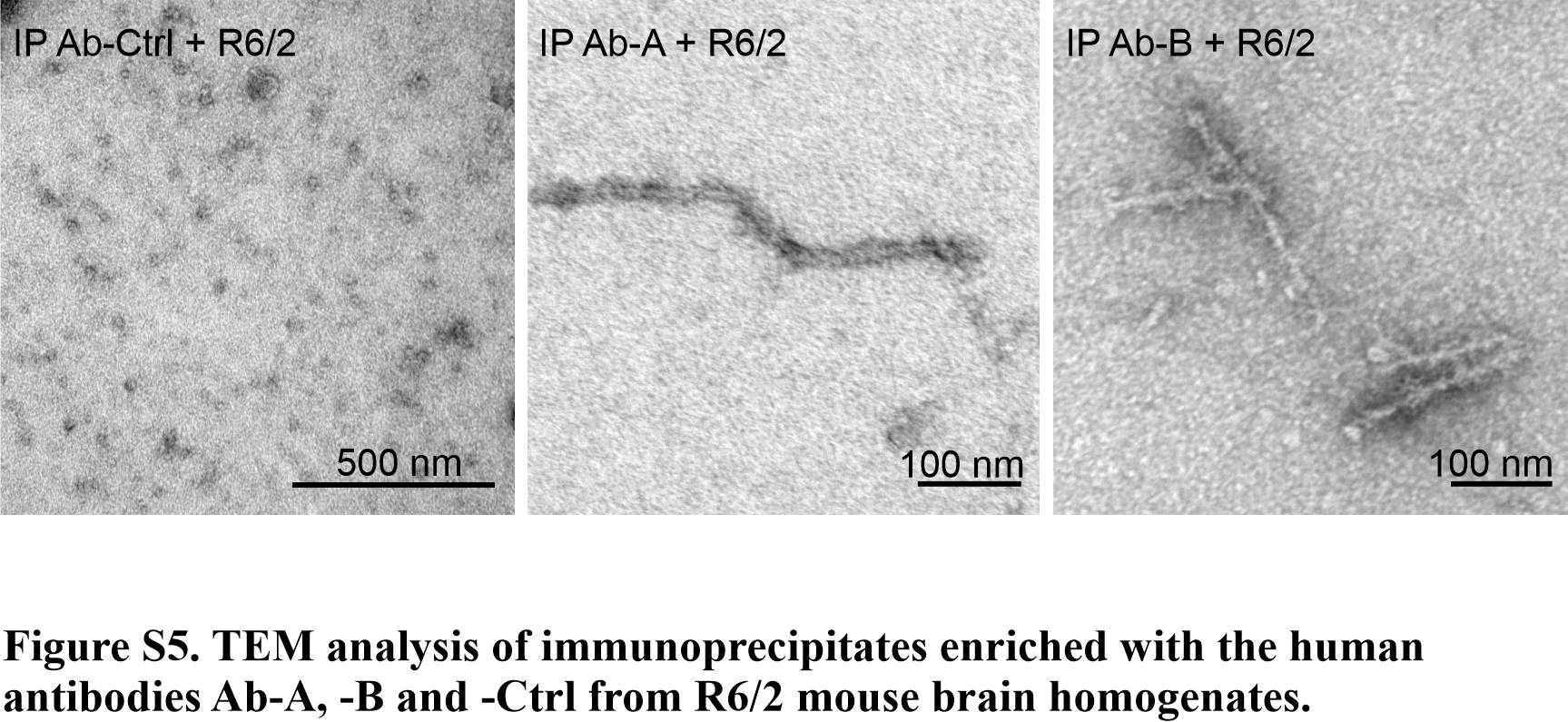

Supplement: Supplementary file 5 [file Image_5.TIF]
