# Supplementary material for: Small, Seeding-Competent Huntingtin Fibrils Are Prominent Aggregate Species in Brains of zQ175 Huntington’s Disease Knock-in Mice
Source: Front Neurosci. 2021 Jun 22;15:682172. doi: 10.3389/fnins.2021.682172 (PMC8257939; doi:10.3389/fnins.2021.682172)
Supplement: Supplementary file 6 [file Table_1.DOCX]

**Table S1. Anti-HTT antibodies**

| **Antibody** | **Description** | **Immunogen** | **Source** | **Reference** |
| --- | --- | --- | --- | --- |
| aAgg | Polyclonal rabbit | HDex1-Q51 fibrils | Own production | Scherzinger et al., 1999 |
| MW1 | Monoclonal mouse | DRPLA-19Q | DSHB, MW1 | Ko et al., 2001 |
| MW8 | Monoclonal mouse | HD exon-1 67Q (soluble 1st and boost with aggregate) | DSHB, MW8 | Ko et al., 2001 |
| MAB5492 | Monoclonal mouse | Recombinant human huntingtin, amino acids 1-82. | MerckMillipore, MAB5492 |  |
| MAB2166 | Monoclonal mouse | Huntingtin fragment from a.a. 181 to 810 as a fusion protein | MerckMillipore, MAB2166 |  |
| MAB2170 | Monoclonal mouse | Huntingtin fragment from aa 1247 to 1646 as a fusion protein | MerckMillipore, MAB2170 |  |
| PHP1 | Monoclonal mouse | GST-tagged recombinant exon1 of wild-type human huntingtin HTTx1 (20Q) protein | Ali Koshnan | Ko et al., 2018 |
| PHP2 | Monoclonal mouse | GST-tagged recombinant exon1 of wild-type human huntingtin HTTx1 (20Q) protein | Ali Koshnan/ MerckMillipore, MABN2450 | Ko et al., 2018 |
| PHP3 | Monoclonal mouse | GST-tagged recombinant exon1 of wild-type human huntingtin HTTx1 (20Q) protein | Ali Koshnan | Ko et al., 2018 |
| PHP4 | Monoclonal mouse | GST-tagged recombinant exon1 of wild-type human huntingtin HTTx1 (20Q) protein | Ali Koshnan | Ko et al., 2018 |
| S830 | Sheep polyclonal | Exon1 of Htt with 53Q | Gillian Bates | (Sathasivam et al., 2013) |
| Ab-A | Monoclonal human IgG1 |  | Neurimmune |  |
| Ab-B | Monoclonal human IgG1 |  | Neurimmune |  |
| Ab-Ctrl | Monoclonal human IgG1 |  | Neurimmune |  |
